# Depletion of Nesprin-2 is associated with an embryonic lethal phenotype in mice

**DOI:** 10.1080/19491034.2018.1523664

**Published:** 2018-09-17

**Authors:** Carmen Mroß, Marija Marko, Martina Munck, Gernot Glöckner, Susanne Motameny, Janine Altmüller, Angelika A. Noegel, Ludwig Eichinger, Vivek S. Peche, Sascha Neumann

**Affiliations:** a Institute of Biochemistry I, Medical Faculty, University Hospital Cologne; Center for Molecular Medicine Cologne (CMMC) and Cologne Cluster on Cellular Stress Responses in Aging-Associated Diseases (CECAD), Koeln, Germany; b Cologne Center for Genomics (CCG), University of Cologne, Koeln, Germany

**Keywords:** Embryonic lethality, leaky expression, ROSA26 locus, mouse embryonic fibroblasts, LINC complex, cell motility, single cell RNAseq

## Abstract

Nesprin-2 is a nuclear envelope component and provides a link between cytoskeletal components of the cytoplasm and the nucleoplasm. Several isoforms are generated from its gene *Syne2*. Loss of the largest isoform Nesprin-2 Giant in mice is associated with a skin phenotype and altered wound healing, loss of C-terminal isoforms in mice leads to cardiomyopathies and neurological defects. Here we attempted to establish mice with an inducible knockout of all Nesprin-2 isoforms by inserting shRNA encoding sequences targeting the N- and C-terminus into the ROSA26 locus of mice. This caused early embryonic death of the animals harboring the mutant allele, which was presumably due to leaky expression of the shRNAs. Mutant embryos were only observed before E13. They had an altered appearance and were smaller in size than their wild type littermates. From this we conclude that the Nesprin-2 gene function is crucial during embryonic growth, differentiation and organogenesis.

## Introduction

The nucleus of eukaryotic cells is separated from the cytoplasm by a double membrane, the outer nuclear membrane (ONM) and the inner nuclear membrane (INM), enclosing the perinuclear space (PNS). Together they form the nuclear envelope (NE). The NE is supported by the lamina, consisting of LaminA/C, LaminB1 and lamina-associated proteins (LAP). INM and ONM are distinguished by specific sets of proteins that provide links to the cytoplasm and the nucleoplasm, respectively. Nesprins, spectrin repeat containing proteins of the NE, are a family of proteins located primarily in the ONM, but are also components of the INM [1,2]. They vary in their domain composition, but all harbor a conserved KASH (Klarsicht-, ANC-, Syne-homology) domain which is responsible for insertion into the nuclear membrane. Through their KASH-domain Nesprins can form a complex, the LINC (linker of nucleoskeleton and cytoskeleton) complex, by binding to the C-terminus of SUN (Sad1p, UNC-84) domain containing proteins in the PNS [3–5]. SUN proteins are components of the INM and interact with lamins, Emerin, components of the nuclear pore complex (NPC) and with chromatin in the nucleoplasm [6–9].

Four different Nesprins are known in human. They differ in size and domain composition and are encoded by separate genes (*SYNE1-4*). Furthermore, the *SYNE1-4* genes give rise to a multitude of isoforms [10,11]. The presence of specific domains is responsible for their linkage to individual cytoskeletal elements, the actin filaments, microtubules or intermediate filaments [2,12–14]. The largest isoform of Nesprin-2, also known as NUANCE, is an ~ 800 kDa protein (6,885 amino acids) with an N-terminal F-actin binding domain (ABD), a rod domain composed of spectrin repeats and the C-terminal KASH domain[2]. Knockdown studies using shRNAs and overexpression of a shortened protein consisting of the ABD, C-terminal spectrin repeats and the KASH domain revealed that Nesprin-2 is responsible for nuclear size, shape and stability, and determines cell architecture and cell polarization [15–17]. Nesprin-2 is also a component of signaling platforms. It interacts with α- and β-catenin and with Emerin and regulates WNT-signaling[18]. A short KASH-less isoform interacts with ERK1/2 kinases at PMLN bodies (promyelocytic leukemia protein nuclear bodies) in the nucleus and enhances ERK1/2 nuclear signaling as shown by increased SP1 activity and ELK1 phosphorylation[19].

Data from the organismal side underline the importance of Nesprin-2. In human, mutations in the *SYNE2* gene cause Emery-Dreifuss muscular dystrophy (EDMD) 5, an autosomal dominant disease[20]. Patients suffering from EDMD harbored a heterozygous DNA variation in the *SYNE2* gene leading to a missense mutation pT89M in the C-terminal Nesprin-2β isoform. This residue is located in a region of Nesprin-2, which mediates Emerin and LaminA/C interactions [6,21,22]. The mutation led to nuclear deformation and LaminA/C, Emerin and SUN2 mislocalization and altered heterochromatin localization in patient fibroblasts[20].

For the mouse *Syne2* gene several mutants have been described, in which the gene was altered by targeted mutation. In one report the N-terminal region of the gene was targeted, and in the three further cases C-terminal regions were targeted. Analysis of knockout animals, in which the ABD of Nesprin-2 was targeted, revealed the importance of Nesprin-2 for the skin. These mice had an increased epidermal thickness, and wound healing experiments showed a delayed closure of the wound underlining its importance in this organ[23]. Targeting of the Syne2 region near the C-terminus in combination with loss of Syne1 C-terminal isoforms in a cardiomyocyte specific fashion led to early onset cardiomyopathy[24]. Investigation of the role of KASH-domain containing Nesprin-1 and Nesprin-2 isoforms in positioning of nuclei in muscle fibers revealed an impact of Nesprin-1 but not of Nesprin-2. A double knockout of KASH-domain containing Nesprin-1 and Nesprin-2 isoforms led to neonatal lethality due to respiratory defects[25]. Later work showed that these mice had brain defects resembling those of SUN1/2 double knockout mice. These defects resulted from a depletion of neural progenitor cells. Nesprin-2 is thought to couple the LINC complex to the centrosome and this link has an impact on the crucial functions of the centrosome during brain development [16,26]. The loss of KASH-domain containing Syne*2* isoforms also affected nuclear migration in photoreceptor cells and caused a reduced thickness of the outer nuclear layer in the eye[27]. In agreement with this result, a mutation was identified in the *Syne2* gene in a mouse mutant with an abnormal cone electroretinography (ERG) response pattern[28]. The mutation was located at position 13,978C> T and led to a stop codon at p.Q4,660* located further upstream of the KASH domain.

So far, a full Nesprin-2 knockout leading to the loss of all Nesprin-2 isoforms and allowing to study its consequences has not been reported. Here we describe our attempts to generate such a full Nesprin-2 knockout using doxycycline inducible shRNA expression targeting N- and C-terminal sequences. Since viable mice carrying the mutated allele were never born we investigated embryos before birth and found that they died at embryonic day 12 at the latest. Analysis of the embryos revealed several defects. Data obtained from the analysis of primary embryonic fibroblasts are in accordance with the proposed role of Nesprin-2 in nuclear integrity.

## Results

### Generation of Nesprin-2 knockdown mice

**Figure 1.**
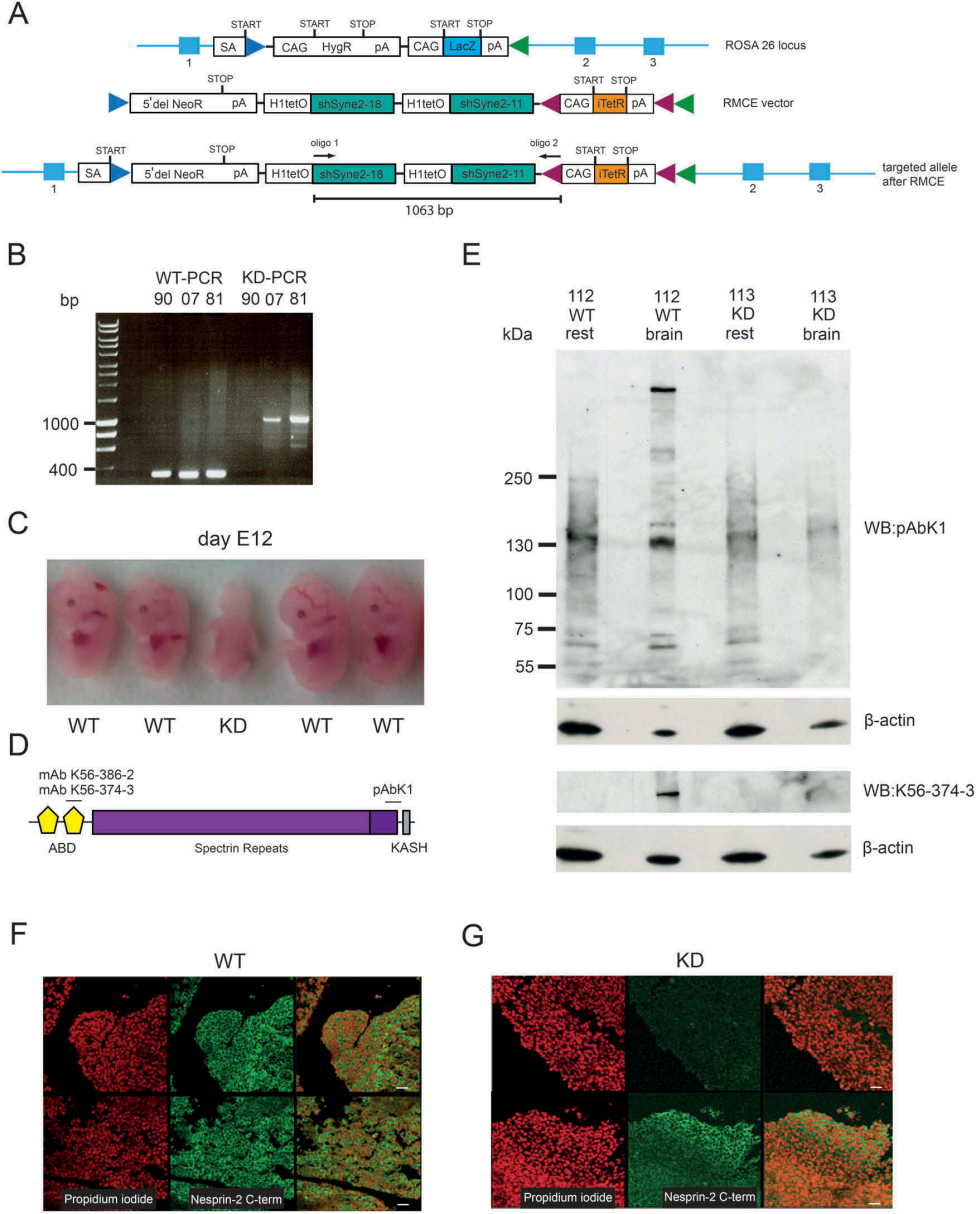
Generation and analysis of Nesprin-2 knockdown animals. (a) Strategy for an N- and C-terminus targeted knockdown of Nesprin-2. The ROSA26 locus is shown, the RMCE vector and the targeted allele after insertion of the vector. CAG, CAG promoter; SA, splice acceptor site; HygR, hygromycin resistance gene; LacZ, β-galactosidase; NeoR, aminoglycoside phosphotransferase gene; pA, polyadenylation site; itetR, improved tetracacycline repressor; H1tetO, H1 promoter and tetracyline operator; shRNA, small hairpin RNA; green arrowhead, FRT site; blue arrow head, F3 site; red arrowhead, loxP site. (b) Genotyping PCRs for embryos 90, 07, 81. Specific primers led to products of 335 bp for the wild type and 1,063 bp for the transgene (see A for the location). (c) Embryos of a wild type female and a chimera at day E12. The genotype indicated below the embryos was determined by PCR. (d) Schematic showing the localization of the epitopes recognized by the Nesprin-2 specific antibodies used in this study. (e) Presence of Nesprin-2 at 796 kDa in lysates from brain and the rest of the embryos as detected with pAbK1. β-actin served as loading control. The lysates were loaded onto a gradient gel (3–12% acrylamide). (f, g) Nesprin-2 expression in tissue from a wild type (WT, F) and a knockdown (KD (KD81), G) embryo. Immunohistochemical staining of paraffin-fixed embryos (E12) is shown. Nesprin-2 was detected with pAbK1 (Nesprin-2 C-term), DNA was stained with propidium iodide. Size bars, 30 µm.

Aim of this study was to generate mice with a doxycycline inducible shRNA mediated knockdown of all Nesprin-2 isoforms. For this, two shRNAs were inserted into the ROSA26 gene locus using a recombinase mediated cassette exchange (RMCE) vector. After the recombinase mediated cassette exchange had taken place, an inducible knockdown allele was present in the ROSA26 locus (Figure 1(a)). Chimeric mice from several ES cell clones were obtained. Since all attempts failed to isolate mice carrying the mutant allele, we thought of the possibility of expression of the shRNAs in the absence of doxycycline in the embryos. Therefore, female mice were bred with male chimeric mice, dissected between day 8 and 13 of pregnancy and the embryos genotyped and used for further analysis. Of 152 successfully genotyped embryos only eight embryos (5.2%) were positive for the mutant allele (Figure 1(b)). All of them were identified among the E8-E12 embryos whereas among the E13 embryos none carried the mutant allele. Embryos carrying the mutant allele were often very small (five embryos), had no eyes and had, in comparison to their littermates, underdeveloped brain and limbs (Figure 1(c), 12 day old embryos; Figure S1, 9 day old embryos). Four were rather fragile and had an altered consistency and nearly fell apart when touched with tweezers (not shown).

In cell lysates from wild type embryos we detected Nesprin-2 Giant (~ 800 kDa) in the brain sample with antibodies against the N- and C-terminal region of Nesprin-2 (Schematic in Figure 1(d)). In the rest of the body no signal was seen (Figure 1(e)). This is in agreement with data from RNA in situ hybridization in which high expression of Nesprin-2 was observed at E14 in the brain and low expression in some parts of the body (http://www.eurexpress.org/ee/). The brain sample from a knockdown embryo did not give a signal. Also, the rest of the body was negative for Nesprin-2 (Figure 1(e)). In immunohistochemical analysis of WT embryonic brain tissue we observed a homogenous staining, whereas with the mutant embryo some areas were completely devoid of staining whereas other areas exhibited Nesprin-2 staining (Figure 1(f,g)).

### Loss of Nesprin-2 affects nuclear envelope proteins

**Figure 2.**
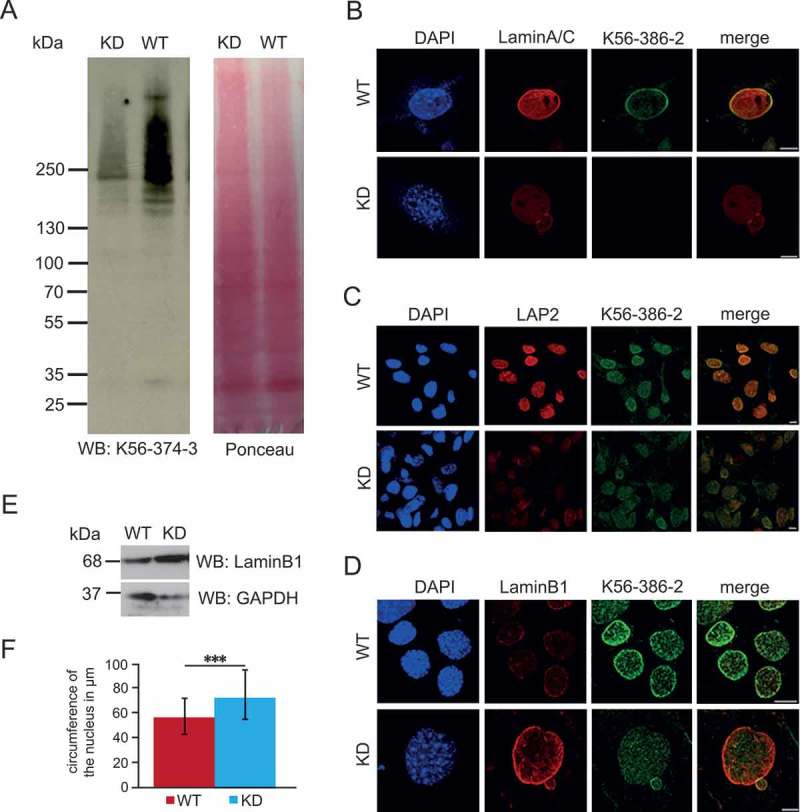
Characterization of nuclei from WT and KD MEFs. (a) Western blot analysis of whole cell lysates from WT144 and KD152 MEFs. The proteins were separated in a gradient gel (3–12% acrylamide). Detection of Nesprin-2 at 796 kDa was with mAb K56-374–3. The corresponding PonceauS stained membrane is shown. (b) LaminA/C staining of WT (WT142) and KD (KD151) MEFs. (c) LAP2 staining of WT (WT134) and KD (KD151) MEFs. The LAP2 antibody recognized all LAP2 isoforms. (d) LaminB1 staining of WT (WT134) and KD (KD151) MEFs. Nesprin-2 was detected with mAb K56-386–2, DNA staining was with DAPI; bars, 10 µM (B-D). (e) LaminB1 upregulation in KD MEFs as detected by western blot analysis. GAPDH levels served as loading control. (f) Circumference of WT and KD nuclei. For the KD (KD151 and 147), only those nuclei from KD cells were taken into account which did not display NE staining by mAb K56-374–3. 66 WT and 65 KD nuclei were measured. ***P < 0.0001.

For studying the impact of the loss of Nesprin-2 at the cellular level we used primary fibroblasts (MEFs) isolated from WT and KD mouse embryos. Western blots revealed the presence of Nesprin-2 Giant at ~ 800 kDa in WT MEFs. Detection was with mAb K56-374–3 which was directed against the ABD of the protein. This band was absent in lysates obtained from KD MEFs (Figure 2(a)). In immunofluorescence experiments mAb K56-386–2 stained the NE in WT MEFs whereas in mutant fibroblasts staining was generally absent (Figure 2(b)). However, with increasing passage numbers K56-386–2 positive cells appeared in the mutant lines. This might be due to the fact that cells, that were not knockdown, overgrew those with a knockdown. Exposure of MEFs to doxycycline, which induced the production of the shRNAs, led to reduced Nesprin-2 levels (Figure S2).

Next, we focused on the nucleus and the NE. For this we used a set of antibodies directed against NE components. We found that LaminA/C labeling was less pronounced in the KD cells (Figure 2(b)). However, when we determined the intensity, the differences were not significant (65 WT cells and 69 KD cells analyzed). Reduced staining was also observed for SUN2, LAP2 (lamina-associated-polypeptide 2) and nuclear pore complex protein NUP107 (Figure 2(c) and data not shown). By contrast, LaminB1 staining was strongly enhanced and the intensities were significantly different (29 WT cells and 16 KD cells analyzed; P, 6.71614E-06). The enhanced levels were confirmed by western blotting (Figure 2(d,e)). SUN1 was neither detected in WT nor in KD MEFs. This is in agreement with results from generally available data bases which indicated a very low expression of SUN1 during embryogenesis.

We also observed that the size of the nuclei in KD fibroblasts was altered. Measurement of the circumference followed by statistical evaluation revealed a significant difference (Figure 2(f)). Furthermore, KD nuclei were often misshapen and exhibited blebs or nuclear lobules (see Figure 2(d)). For WT, 36% of cells had deformed nuclei, for KD cells we observed 42% with deformed nuclei (152 WT cells and 91 KD cells analyzed).

### DNA damage in Nesprin-2 deficient MEFs

**Figure 3.**
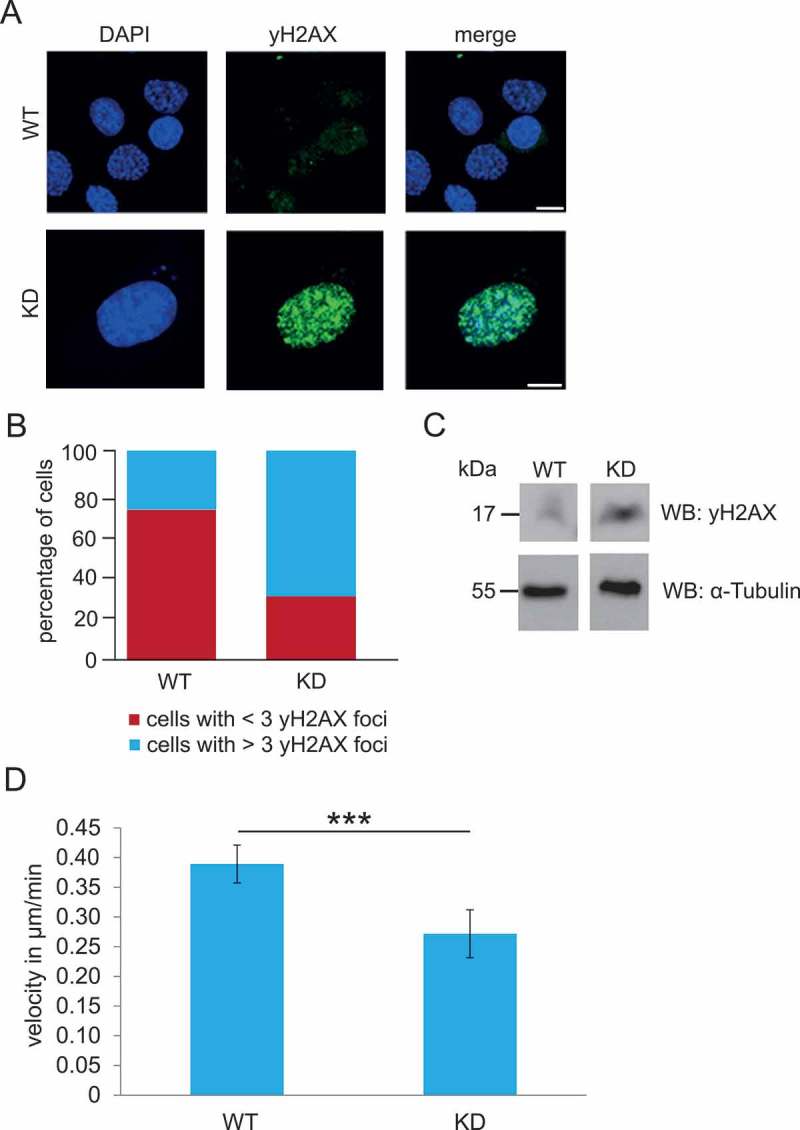
DNA damage and cell migration in WT and KD MEFs. (a) γH2AX staining as an indication of DNA damage of WT (WT134) and KD (KD152) MEFs. Bar, 10 µM. (b) Evaluation of cells with < 3 and > 3 γH2AX positive foci per nucleus. For WT 277 cells and for KD 246 cells were evaluated. (c) Western blots of cell lysates from WT (WT134) and KD (KD147) cells probed for γH2AX and α-tubulin for control. The data are from a single blot, however the lanes shown were not adjacent to each other. (d) Velocity of WT (WT134) and KD (KD151) cells in µm/min. ***P, < 0.0001.

Proteins associated with the NE have an important role for the genome organization and integrity, and structural abnormalities of the NE have been associated with DNA repair defects. This is particularly well studied in Hutchison-Gilford-Progeria Syndrome (HGPS) where an impaired repair of DNA double strand breaks (DSB) correlates with abnormal nuclear morphology[29]. Also, in Nesprin-1 depleted cells and tumor cells exhibiting low levels of Nesprin-1 we found increased numbers of γH2AX foci indicating elevated levels of DNA damage[30]. Histone H2AX is a key component in DNA repair. It is phosphorylated in response to DNA damage and binds to DNA double strand breaks. In WT MEFs we did not find γH2AX foci in the majority of cells, whereas many KD MEFs harbored γH2AX foci. For quantification we determined the number of γH2AX positive cells with less than 3 or 3 foci (≤ 3) and cells with more than 3 foci (> 3) and found that 76.17% of WT (277 cells analyzed) and 31.71% of KD MEFs (246 cells analyzed) had ≤ 3 foci, and 23.83% of WT and 68.29% of KD MEFs had > 3 foci (Figure 3(a,b)). This increase in the number of γH2AX foci in the KD was also seen in western blots (Figure 3(c)).

### Cell proliferation and cell migration

Cell proliferation was followed in two independent experiments over 120 hours. The duplication time for wild type MEFs was 44.15 ± 4.5 h and for the KD 42.1 ± 2.55 h and thus was not significantly different.

Previous work with MEFs or keratinocytes defective for Nesprin-2 Giant had revealed a slower migration [15,23]. We performed migration assays with WT134 and KD151 cells using culture plates with specific inserts that were removed 24 hours after plating in order to create a gap into which cells could migrate. Tracking of the cells was carried out from 2 till 10 hours and 45 minutes and the migration of single cells followed. All together four independent experiments were carried out and 120 cells per experiment for wild type and knockdown each analyzed. The analysis revealed that WT cells migrated at a speed of 0.39 µm per minute whereas for KD cells the speed was significantly reduced to 0.27 µm per minute (P < 0.0001) (Figure 3(d)). Cell polarity did not appear to be affected in the KD cells. They migrated in a directed fashion and, as revealed by pericentrin staining, had their centrosome reoriented towards the front of the cells (data not shown).

### Single-cell RNA-sequencing

**Figure 4.**
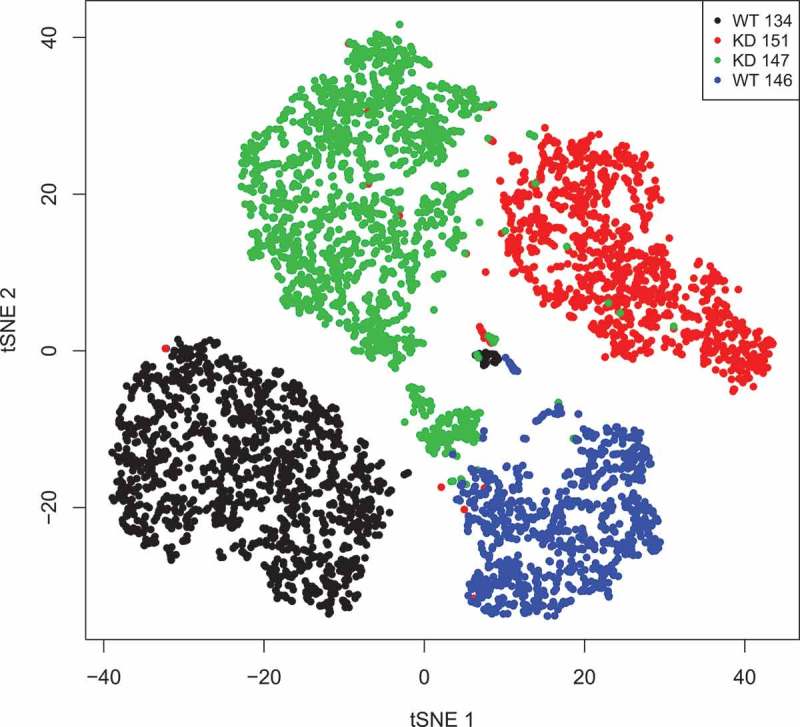
t-SNE blot of the RNAseq data from WT and KD cells. t-SNE blot of the four samples (tSNE 1 vs tSNE 2) based on the normalized expression value for each cell. Each dot represents one cell in the respective sample.

In recent years high-throughput single-cell RNA-sequencing (scRNA-seq) has become available. We employed this method in order to obtain an insight why loss of Nesprin-2 was detrimental to the embryo. We used MEFs from two wild type (WT134 and WT146, passage number 9 and 13, respectively) and two mutant embryos (KD147 and KD151, passage number 13 and 16, respectively). A total of 1,548 (WT134) and 927 (WT146) wild type MEFs and 1,758 (KD147) and 1,186 (KD151) mutant MEFs were used for scRNA-seq and 40 Gb of raw data were generated with approximately 50,000 reads per library and between 600 and 1,000 genes per cell quantified (Figure 4). Among the KD147 cells we detected Nesprin-2 transcripts in 219 cells (12.45%) and for KD151 in 246 cells (~ 20%). Thus, only a minor fraction of cells has detectable Nesprin-2 expression levels. Surprisingly, for the WT MEFs similar numbers were obtained. For WT134 280 out of 1,548 cells (18.6%) and for WT146 103 out of 927 cells (11.1%) were Nesprin-2 positive, possibly caused by the repeated passage of lines.

For subsequent analysis we used the data from Nesprin-2 positive WT134 and WT146 and Nesprin-2 negative KD147 and KD151 MEFs. In this comparison we observed very few significant differences. Gja1 (P, 0.0005), ExoSC7 (P, 0.0005) and Tpm3-RS7 (P, 6,85E-25) were significantly upregulated in the KDs. Tpm3-RS7 was almost exclusively expressed in the KD MEFs. Cdkn2c (P, 0.065) and Gatad1 (P, 0.078) were downregulated. The data are from a comparison of both WTs to both KDs. Gja1 corresponds to Connexin43, ExoSC7 is a non-catalytic component of the RNA exosome complex, Tpm3-RS7 is Tropomyosin3, Cdkn2c is Cyclin Dependent Kinase Inhibitor 2C and inhibits CDK4, and Gatad1 is a zinc finger containing protein that binds to a histone modification site that regulates gene expression. The results were confirmed by quantitative RT-PCR (Table 1). For the transcripts corresponding to LaminA/C, SUN2, NUP107, LAP2 and LaminB1 no differences were observed in this analysis although we had observed significant changes in the immunofluorescence staining for LaminB1.

**Table 1.** Differentially regulated genes in WT and KD MEFs. qRT-PCR was performed for the indicated genes and the up- or downregulation as compared to the WT values determined (fold change).

Fold change	WT134	WT146	KD147	KD151
Gja1	1	−5.48	4.35	1.95
ExoSC7	1	1.83	2.69	1.61
Tpm3-RS7	1	1.47	1.45	2.46
Cdkn2c	1	−4.46	−3.69	−21.27
Gatad1	1	1.12	1.35	−1.2

## Discussion

In this work we attempted to generate an inducible full Nesprin-2 knockout using an shRNA approach by targeting N- and C-terminal regions of the Syne2 gene. The construct was inserted into the ROSA26 locus. The results indicated that the knockdown was leaky so that the shRNAs were expressed without induction. Similar observations have been made by others when using the ROSA26 locus. For instance, a conditional expression construct for diphtheria toxin inserted into the ROSA26 locus, which could be activated through Cre-mediated recombination, resulted in homozygous mutant mice with degenerative abnormalities in several tissues without induction of the Cre-recombinase due to leaky expression of the construct[31]. Related findings were reported by Sinha and Lowell (2017) who observed a significant leakiness without doxycycline induction in a system using the ROSA26 locus in different mice strains[32].

Leakiness may indeed be the cause of early embryonic death in case of our Nesprin-2 KD mice. Early embryonic death is often a phenotype that occurs in mice carrying genetically engineered mutations. It is a sign that the corresponding gene is required at a particular stage during embryogenesis[33]. In order to detect mutant embryos we dissected mice from embryonic day E8 onward and identified embryos carrying the Nesprin-2 knockdown cassette only from E8 until midgestation (E12). This is the time when organogenesis takes place. At later time points all embryos were wild type. As the number of mutants was very low (8 out of 152 embryos analyzed) we presume that mutants died earlier than E8 and that only those embryos survived, in which the expression of the shRNAs was at such a level which may have allowed some Nesprin-2 production in order to survive up to these stages. We conclude that Nesprin-2 has a fundamental role in embryonic development. Nevertheless, our data are surprising in view of the various mouse models in which specific isoforms were targeted and which did not lead to early embryonic death. One reason could be that isoforms that are still produced in these mice can take over functions. This is no longer possible in a full knockout. A detailed analysis of Nesprin-2 isoforms during embryogenesis could help to solve this question.

Staining of MEFs derived from mutant embryos showed a mixture of Nesprin-2 negative and positive cells. The latter increased in number with increasing passage number. The analysis of Nesprin-2 negative MEFs revealed that they had similar phenotypes as has been described previously for MEFs isolated from Nesprin-2 mouse mutants and for cells in which Nesprin-2 was depleted by shRNAs [15,20,23]. In particular, changes in the nuclear morphology and in cell migration resembled those that had been seen previously.

Primary mouse embryonic fibroblasts are considered to be a homogenous cell type. A recent study however identified high developmental and phenotypic diversity between the cells stemming from a single animal with single cells showing individual transcriptional profiles[34]. This could explain why our scRNA-seq analysis resulted in the identification of only a few candidate genes whose differences in expression rose to statistical significance when we compared transcriptomes of cells from two Nesprin-2 KD embryos to those of two WT embryos.

A surprising finding from the scRNA-seq analysis was the absence of Nesprin-2 transcripts from nearly as many WT as KD cells despite the presence of Nesprin-2 at the NE in WT MEFs. An explanation could be the stability of the protein influencing its levels in individual cells. Also, the expression of the Syne2 gene during embryogenesis is not known. We found that the gene is transcribed in ES cells, further data are then only known for E14 stage embryos where the mRNA was detected at low levels in the body of the embryo and at higher levels in brain (http://www.eurexpress.org/ee/).

For our qPCR experiments we isolated total cellular RNA from a pool of cells for reverse transcription. This does not allow the analysis of transcripts form Nesprin-2 KD cells with shRNA expression exclusively. However, we could confirm upregulation of Gja1 in both KDs, of ExoSC7 in KD147 and Tpm3-RS7 in KD151 in qPCR experiments. Gja1 (Connexin43) upregulation could lead to a decreased cell migration, as we have observed for KD fibroblasts (Figure 3(d)) and further on to defective organogenesis, in particular heart development[35]. Changes in ExoSC7 and Tpm3-RS7 expression in early developmental stages point to compromised RNA processing and degradation and a disorganized cytoskeleton based on the function of Tropomyosin in striated muscle, respectively, that could result in or contribute to the observed embryonic lethality.

## Materials and methods

### Generation of Nesprin-2 knockdown mice

For creating a Nesprin-2 knockdown, an inducible RNAi approach via targeted transgenesis of two shRNA cassettes in the ROSA26 locus was chosen [36–38]. The ROSA26 locus was equipped with RMCE (Recombinase-Mediated Cassette Exchange) docking sites. The two shRNAs were specific for the N-terminus (siSyne2_18, 5ʹ GAGGCACAGATCCAACAAA 3ʹ, position 914–932, exon 8; the full length sequence inserted in the shRNA vector was 5ʹ TCCC GAGGCACAGATCCAACAAA TTCAAGAGA TTTGTTGGATCTGTGCCTC TTTTTA) and for the C-terminus (siSyne2_11, 5ʹ GCAGCGAGATATCGAACAA 3ʹ, position 18,377–18,395, exon 101; the full length sequence inserted in the shRNA vector was 5ʹ TCCC GCAGCGAGATATCGAACAA TTCAAGAGA TTGTTCGATATCTCGCTGC TTTA), respectively, which should lead to the knockdown of most isoforms. The shRNAs had been tested for their knockdown efficiency in ES cell clones by qRT-PCR. Specifically, individual shRNAs were cloned into the targeting vector allowing inducible expression of the shRNA. The vector was integrated into the defined locus, targeted cells were isolated and validated, and shRNA expression was induced. Total RNA was prepared from ES cells and used for qRT-PCR. Tests for off-target effects on Syne1, Syne3 and Syne4 expression in ES cells were negative. Finally, the selected sequences were cloned into an RMCE vector which integrated in the ROSA26 locus. All this work, including the mouse generation (C57BL/6 background) was carried out by Taconic (Taconic Biosciences, GmbH).

For genotyping of mice harboring the Nesprin-2 knockdown sequences in the ROSA26 locus, primer pair forward (oligo 1) 5ʹGTTGGGTCCACTCAGTAGATGCC3ʹ and reverse (oligo 2) 5ʹGGAACATACGTCATTATTGACGTC3ʹ resulting in a 1063 bp product or Syne2Fp1 forward 5ʹCCATGGAATTCGAACGCTGAC3ʹ and Syne2TRp3 reverse 5ʹGGAACATACGTCATTATTGACGTC3ʹ resulting in a 901 bp product were used for PCR analysis. For the DNA control, forward primer 5ʹGTGGCACGGAACTTCTAGTC3ʹ and reverse primer 5ʹCTTGTCAAGTAGCAGGAAGA3ʹ amplified a product of 335 bp. Annealing was done at 62°C and 45 seconds for the mutant primers, and 60°C and 30 seconds for the wild type primers. 36 and 35 cycles, respectively, were performed. Embryos 07, 81, 114, 131, 147, 149, 151, and 152 carried the knockdown allele. All together 152 embryos were genotyped.

All animal experiments were performed according to institutional guidelines and an animal license of the State Office of North-Rhine-Westphalia, Germany.

### Dissection of mice and embryo isolation

Female mice were kept with the male chimeras and plug checked. Mice at day 8–13 of the pregnancy were used. The complete embryonic sack chain was isolated and washed with PBS. The embryonic sacks were opened carefully in fresh PBS and the embryos isolated and photographed. The embryos were decapitated, a piece of the tail was taken for genotyping and the brain and the rest of the body collected for preparation of lysates.

### Establishment of primary fibroblasts from mouse embryos

Primary fibroblasts from mouse embryos (MEFs) were isolated at day E8-E12 as described[39]. Briefly, embryos were isolated from the uterus, rinsed with PBS, and a small tail biopsy was taken for genotyping. The embryos were transferred to a 1.5 ml tube each containing 300 µl Trypsin/EDTA solution (0.05% Trypsin and 0.02% EDTA in PBS) and pipetted up and down. Incubation was for 35 minutes at 37°C. The reaction was terminated with 600 µl pre-warmed medium and the cell suspension transferred into a 6 cm dish containing 3 ml of medium (DMEM supplemented with 10% FBS, nonessential amino acids, 2 mM L-glutamine and 100 U/ml penicillin and 100 µg/ml streptomycin). The cells were incubated at 37°C and 5% CO_2_ in a cell culture incubator for several days until the cells could be split. For induction of shRNA production, cells were treated with doxycycline (0.25µg/ml) for 76 hours.

### Cell migration assay

Cell migration analysis was performed with MEFs using ibidi microscopy slides with 8 well chambers (ibidi GmbH, Martinsried, Germany) into which culture-inserts were placed in order to generate a gap into which cells could migrate. ~ 50,000 cells were deposited per side of the well[40]. Cell migration was followed by time lapse video microscopy (37°C, 5% CO_2_) using a Leica DMIRE2 microscope. Analysis of the data was with Image J ‘Manual Tracking’ and ‘Chemotaxis tool’[39].

### Immunofluorescence analysis, antibodies and analysis of nuclei

For immunofluorescence analysis, fixation of cells was with ice cold methanol or paraformaldehyde (PFA) depending on the antibody. The following antibodies were used: mouse monoclonal antibody (mAb) K56-374–3 and mAb K56-386–2 against Nesprin-2 N-terminal region; pAbK1, polyclonal rabbit antibodies recognizing C-terminal sequences of Nesprin-2; rabbit pAb LaminB1 (Abcam), mAb against Emerin (Abcam); mAb K3-184–2 specific for GFP; mAb against GAPDH (Sigma); rabbit pAb LAP2 (Abcam); mAb NUP153 (Abcam); goat pAb NUP107 (Santa Cruz biotechnology); rabbit pAb LaminA/C (Santa Cruz biotechnology); rat mAb YL1/2 against α-tubulin; pAb SUN1 (Abcam); pAb SUN2 (Abcam); mAb γH2AX (Millipore); pAb HP1β (Sigma). Highly preabsorbed and affinity purified Alexa 488 and Alexa 568 conjugated secondary antibodies (Molecular Probes) were used in indirect immunofluorescence. 4,6-diamino-2-phenylindone (DAPI, Sigma) was used to visualize DNA [15,21,41,42]. Appropriate controls with secondary antibodies only were performed. Staining intensities were determined using Leica software. Low passage numbers of the cells were used for the analysis. For evaluation of KD cells only cells with low or no nuclear Nesprin-2 staining as revealed by mAb K56-386–2 were used. The circumference of nuclei of cells as revealed by DAPI staining was marked using the LASAF software of the Leica microscope. The software showed the µm values for the circumference. Excel was used for the statistical analysis.

### Immunohistochemistry

For immunohistochemistry, head and body of the embryos were transferred into a fixation chamber and embedded in paraffin and processed as described[43]. Detection of Nesprin-2 was with pAbK1, DNA was stained with propidium iodide (Sigma-Aldrich).

### Single-cell RNA-seq, data processing and analysis

For single-cell RNA-sequencing (scRNA-seq) primary embryonic fibroblasts from WT134 and WT146 and KD147 and KD151 were used. For WT134 1,548 cells, for WT146 927 cells, for KD147 1,758 cells and for KD151 1,186 cells were analyzed. The experiments were performed at the Cologne Center for Genomics (CCG).

### Droplet-based scRNA-seq

Single cells were counted manually and resuspended into 0.04% BSA-PBS. The cellular suspensions were processed through the GemCode Single Cell Platform using the GemCode Gel Bead, Chip and Library Kits (10x Genomics, Pleasanton, USA) according to the manufacturers protocol. About 3,000 cells for each sample were loaded into four channels of the chromium system with an average recovery rate of 1,500 cells. Cells were captured and lysed and barcoded reverse transcription of RNA was performed, followed by library preparation. All samples were multiplexed together and sequenced across one lane of an Illumina HiSeq 4000 sequencing system with the first read analyzing the single cell index and the second read of 100 nt addressing the 3ʹ mRNA transcriptome. The Cell Ranger Suite was used to perform sample de-multiplexing, barcode processing and single cell gene UMI (unique molecular index) counting.

### Analysis of raw data

Demultiplexing and generation of FastQ files was performed with Illumina’s bcl2fastq2 software (version 2.18.0.12) with automatic trimming of Illumina adapters enabled. The FastQ files were further processed with the 10x Genomics cellranger pipeline (version 2.0.1). In particular, read counts per gene were generated for each of the four samples individually (cellranger count) using mm10 as the reference and 1,500 as the expected number of cells. Afterwards, the data from the four samples were aggregated (cellranger aggr) to compute normalized expression values and perform clustering using default parameters. Loupe cell browser (10x Genomics) was used for data visualization and gene expression analysis.

### RNA isolation, cDNA synthesis and verification of RNA-seq data with qPCR

Total cellular RNA was isolated with RNeasy mini kit (Qiagen) and treated with DNaseI according to manufacturer’s instructions. For the cDNA synthesis M-MLV reverse transcriptase (H-) (Promega) and random primers pdN6 (Roche) were used (RT+). In order to assess possible contamination with genomic DNA in subsequent qRT-PCR experiments, mock reverse transcription reactions lacking the enzyme (RT-) were performed in parallel.

Quantitative real-time PCR experiments were performed with StepOnePlus™ Real-Time PCR cycler (Applied Biosystems, CA, USA), reactions were prepared with Luna® Universal qPCR Master Mix (New England Biolabs) and the data was analyzed with StepOne software (Applied Biosystems, CA, USA). The expression of the GAPDH gene served for normalization.

Following primer pairs were used: Connexin43, 5ʹ-ACAGCGGTTGAGTCAGCTTG-3ʹ and 5ʹ-GAGAGATGGGGAAGGACTTGT-3ʹ; Exosc7, 5ʹ-GTGAGGACTACCGATGTGTTGA-3ʹ and 5ʹ-AGCTTCGGTGTCCCCATTTC-3ʹ; Tpm3-RS7, 5ʹ-TCCAGGTTCTGCAGCAGCAA-3ʹ and 5ʹ-TTCAGCCTGCTTCCGGGC-3ʹ; Gatad1, 5ʹ-TATCAAAGCGCCTGAATCTGTT-3ʹ and 5ʹ-TCTGAGCATAGTACGGCTTCC-3ʹ; Cdkn2c, 5ʹ-GGGGACCTAGAGCAACTTACT-3ʹ and 5ʹ-AAATTGGGATTAGCACCTCTGAG-3ʹ; GAPDH, 5ʹ-AGGTCGGTGTGAACGGATTTG-3ʹ and 5ʹ-TGTAGACCATGTAGTTGAGGTCA-3ʹ;

PCR reactions were first heated for 10 min at 95 °C and then 40 cycles of 15 s at 95 °C and 1 min at 60 °C followed. Melting curve analysis at the end of each DNA amplification run confirmed the specificity of gene amplification. The cycle threshold (Ct) differences between corresponding RT+ and RT- samples were equal or bigger than 10 cycles.

## Miscellaneous methods

For western blot analysis, proteins were extracted from tissue or cells as described [15,39]. Proteins were separated by gradient gel electrophoresis (3–12% or 4–20% acrylamide; Thermo Fisher Scientific) and blotted to nitrocellulose membranes[15]. Statistical evaluation was done using the functions of Microsoft Excel or GraphPad Prism software.
