# Supplementary figures and images for: Depletion of Nesprin-2 is associated with an embryonic lethal phenotype in mice
Source: Nucleus. 2018 Sep 17;9(1):503–15. doi: 10.1080/19491034.2018.1523664 (PMC6244730; doi:10.1080/19491034.2018.1523664)

KD

WT

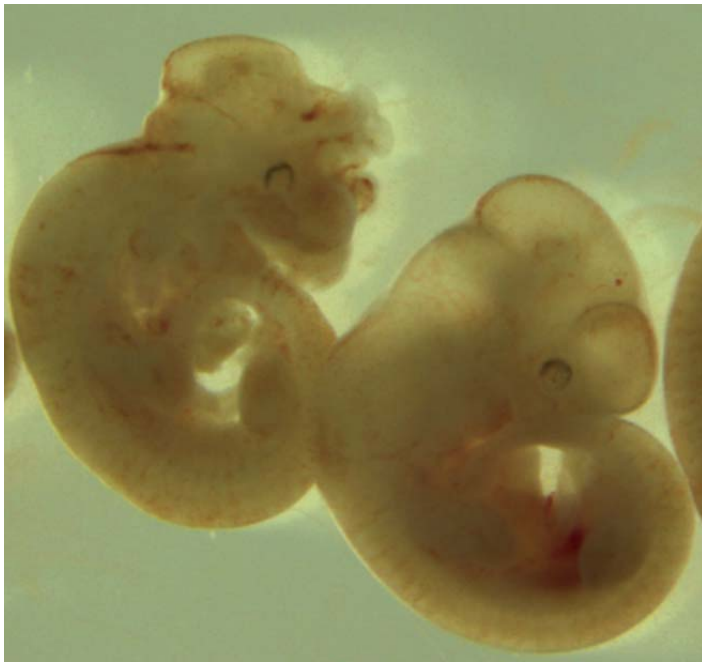

Figure S1

Supplement: Supplemental Material [file kncl-09-01-1523664-s001.zip › Supplementary material/Supplementary figure 1.pdf]

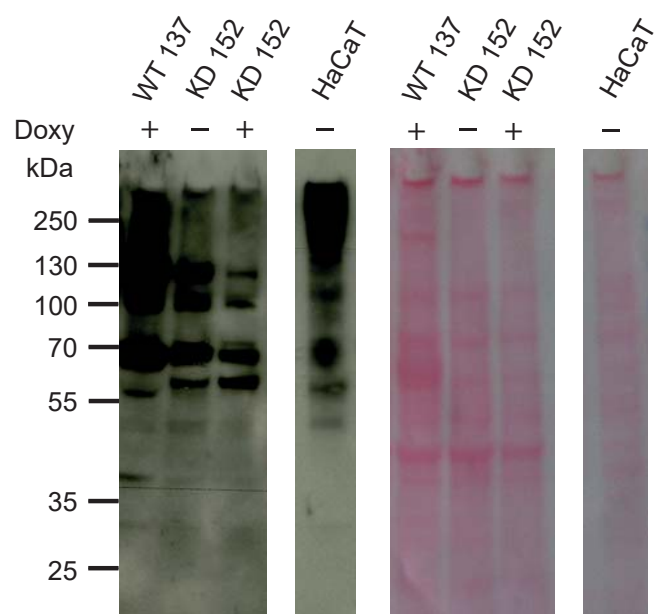

Figure S2

Supplement: Supplemental Material [file kncl-09-01-1523664-s001.zip › Supplementary material/Supplementary figure 2.pdf]
